# Genome-Wide Association Study of Absolute QRS Voltage Identifies Common Variants of TBX3 as Genetic Determinants of Left Ventricular Mass in a Healthy Japanese Population

**DOI:** 10.1371/journal.pone.0155550

**Published:** 2016-05-19

**Authors:** Motoaki Sano, Shigeo Kamitsuji, Naoyuki Kamatani, Yasuharu Tabara, Takahisa Kawaguchi, Fumihiko Matsuda, Hiroyuki Yamagishi, Keiichi Fukuda, Japan Pharmacogenomics Data Science Consortium (JPDSC)

**Affiliations:** 1 Department of Cardiology, Keio University School of Medicine, Tokyo, Japan; 2 StaGen Co. Ltd., Tokyo, Japan; 3 Center for Genomic Medicine, Kyoto University Graduate School of Medicine, Kyoto, Japan; 4 Department of Pediatrics, Keio University School of Medicine, Tokyo, Japan; 5 JPDSC Executive Office (StaGen CO. Ltd.), Tokyo, Japan; Niigata University Graduate School of Medical and Dental Sciences, JAPAN

## Abstract

Left ventricular hypertrophy (LVH) represents a common final pathway leading to heart failure. We have searched for genetic determinants of left ventricular (LV) mass using values for absolute electrocardiographic QRS voltage in a healthy Japanese population. After adjusting for covariates, the corrected S and R wave voltages in leads V1 and V5 from 2,994 healthy volunteers in the Japan Pharmacogenomics Data Science Consortium (JPDSC) database were subjected to a genome-wide association study. Potential associations were validated by an *in silico* replication study using an independent Japanese population obtained from the Nagahama Prospective Genome Cohort for Comprehensive Human Bioscience. We identified a novel association between the lead V5, R wave voltage in Japanese individuals and SNP rs7301743[G], which maps near the gene encoding T-box transcription factor Tbx3. Meta-analysis of two independent Japanese datasets demonstrated a marginally significant association of SNP rs7301743 in TBX3|MED13L with a 0.071 mV (95% CI, 0.038–0.11 mV) shorter R wave amplitude in the V5 lead per minor allele copy (P = 7.635 x 10^−8^). The transcriptional repressor, TBX3, is proposed to suppress the development of working ventricular myocardium. Our findings suggest that genetic variation of Tbx3 is associated with LV mass in a healthy Japanese population.

## Introduction

Left ventricular hypertrophy (LVH) represents a common final pathway leading to heart failure [1], and is associated with an increased vulnerability to sudden death and acute myocardial infarction [2]. Left ventricular (LV) mass can be regarded as a quantitative trait that is affected by both environmental and genetic factors that vary considerably between individuals. The search for genes conferring an individual's genetic susceptibility to LVH is clinically relevant. Early diagnosis through identification of genetic polymorphisms indicative of disease, along with subsequent intervention, has the potential to bring about a new era of preemptive medicine for cardiovascular diseases.

It is not realistic to expect an ECG to diagnose LVH with more than a modest degree of accuracy [3]. Nevertheless, epidemiological research has demonstrated that electrocardiographically diagnosed LVH (ECG-LVH) carries significant prognostic value [4]. Consistent with this, recent genome-wide association studies (GWAS) and genome-wide linkage analysis have shown that ECG-LVH provides unique information about the genetic determinants of LVH [5] [6] [7].

Previous GWAS for ECG-LVH were designed to compare cases of ECG-LVH with controls. Logistic regression tested for associations between SNPs and the case-control status. ECG-LVH is largely dependent on fixed voltage thresholds. Using these measures, individuals with mild LVH may fail to fulfil the criteria for a diagnosis of LVH. Indeed, defining LVH as a qualitative trait using fixed voltage criteria risks losing valuable information contained in the magnitude of QRS voltages.

In this study, we have therefore investigated genetic determinants of LV mass in a healthy Japanese population by a GWAS using absolute QRS voltage.

## Material and Methods

### JPDSC

The Japan Pharmacogenomics Data Science Consortium (JPDSC) is comprised of six pharmaceutical companies: Astellas, Daiichi Sankyo, Mitsubishi Tanabe, Otsuka, Taisho, and Takeda. The JPDSC maintains a database of 2,994 healthy Japanese volunteers for pharmacogenomics (PGx) studies, which contains the genotypes of 2.5 million single-nucleotide polymorphisms (SNPs) and 5 human leukocyte antigen loci per person, as well as other clinical information, such as physiological, haematological, and biochemical data. The data set was obtained in two phases. The first data set was collected between 2000 and 2003, with the remaining data set gathered in a second phase from 10 geographic regions in Japan. Sample collection was validated by the principal component analysis. All subjects gave written informed consent, and we obtained ethical approval for the study from the ethics committee of JPDSC (Ichiro Matsuda, Ryuichi Ida, Yayoi Sasaki, Sumio Sugano, Eiko Suda, Masashi Tokunaga, Toru Masui, Kaori Muto) [8].

### Statistical analysis for GWAS

Genomic DNAs were genotyped on an Illumina Human Omn 2.5.8 BeadChip. SNPs that met the following criteria were used in the GWAS: SNP call rates (≥ 95%), the Hardy-Weinberg equilibrium test (*P* ≥ 0.01), and minor allele frequency (≥ 1%). We used linear regression to adjust ECG measures for independent variables within each phase. In each phase, we combined the standardised residuals and used them as quantitative phenotypes for the association analysis with PLINK (version 1. 1. 3). We evaluated if a trait and genotype were significantly associated using Wald test [9].

### Replications and Meta Analysis

Replication of candidate SNPs was tested using datasets obtained from the Nagahama Prospective Genome Cohort for Comprehensive Human Bioscience (the Nagahama Study [n = 2941]) a community-based prospective multiomics cohort study conducted by Kyoto University. The covariates used for the correction in the replication study included age, sex, log HR, and systolic pressure of participants (i.e. Nagahama city). Genomic DNAs were genotyped on an Affymetrix 5.0 SNP array. We replicated 13 different associations, thus the P-value threshold of 0.05/13 = 0.0038 was used according to Bonferroni’s correction [10]. Meta-analysis was performed using the inverse variance method by using R environment (version 2.15.0).

The JPDSC data (the allele frequency data from about 1.4M SNP of all 2994 Japanese healthy volunteers and each birth place region) and individual genotype data of the Nagahama cohort participants are now available in the National Bioscience Database Center, http://humandbs.biosciencedbc.jp/.

## Results

### Clinical and electrocardiographic characteristics of JPDSC data set

For statistical analysis, we used phase 1 JPDSC dataset of 992 healthy Japanese volunteers (596 males and 396 females) and phase 2 JPDSC dataset of 2,002 healthy Japanese volunteers (998 males and 1,004 females). The mean, median, minimum, and maximum of clinical parameters [age, body mass index (BMI), heart rate, systolic and diastolic blood pressure, serum potassium, and serum calcium] and electrocardiographic LV voltages [RV5 = R wave in V5 (mV), SV1 = S wave in V1 (mV), RV5 + SV1 = the sum of RV5 + SV1 (mV)] are summarized in Table 1.

**Table 1:** Characteristics of participants in JPDSC.

Qualitative Variable	Phase	Levels		Size				
Gender	Phase 1	Male/Female		596/396 (Total 992)				
	Phase 2	Male/Female		998/1004 (Total 2002)				
Quantitative Variable	Phase	Mean	SD	Min.	1st Q.	Median	3rd Q.	Max.
Age (years)	Phase 1	38.76	11.67	20	29	36	46	74
	Phase 2	33.40	9.94	20	26	32	38	75
BMI (kg/m^2^)	Phase 1	22.31	2.00	16	20.22	22	23.9	37.4
	Phase 2	21.94	3.39	14.1	19.6	21.3	23.7	45.1
Systole (mmHg)	Phase 1	127.20	2.00	84	114	124	138	199
	Phase 2	118.70	15.62	77	108	117	128	198
Diastole (mmHg)	Phase 1	78.69	2.00	39	70	77	86	125
	Phase 2	72.38	11.24	40	64	71	80	123
HR (bpm)	Phase 1	74.56	5.00	43	67	74	81	126
	Phase 2	67.18	10.13	34	60	66	73	115
K (mEQ/L)	Phase 1	3.56	1.00	2.5	3.4	3.6	3.7	4.7
	Phase 2	4.11	1.00	3	3.9	4.1	4.3	19.6
Ca (mg/dL)	Phase 1	9.38	1.00	7.9	9.1	9.4	9.6	10.9
	Phase 2	9.48	1.00	7.9	9.2	9.5	9.7	13.4
RV5	Phase 1	1.68	5.00	0.325	1.275	1.62	1.993	3.97
	Phase 2	1.47	0.53	0.03	1.09	1.4	1.75	4.7
SV1	Phase 1	1.05	5.00	0	0.7325	1.01	1.3	3.25
	Phase 2	1.00	1.00	0.05	0.69	0.94	1.25	3.2
RV5+SV1	Phase 1	2.73	6.00	0.75	2.205	2.625	3.205	5.705
	Phase 2	2.47	0.78	0.72	1.91	2.4	2.92	7.55

### Clinical factors accounting for variations in ECG LV voltages

To examine the variation in electrocardiographic LV voltages between individuals, we first used multiple linear regression analysis to evaluate the effects of non-genetic factors (S1 Table). Outliers, defined as values four standard deviations higher or lower than the mean, were removed (S1 Fig).

We tested whether gender, age, log-transformed (log) BMI, log heart rate (HR), systolic pressure, diastolic pressure, serum potassium, serum calcium, and Japanese geographic region should be included as covariates. Based on these preliminary results, we used: age, gender, log HR, and systolic pressure as covariates for log RV5; age, gender, log BMI, and systolic pressure as covariates for log SV1; and age, gender, log BMI, log (HR), and systolic pressure as covariates for log (RV5+SV1).

### Possible novel loci associated with ECG LV voltages

**Fig 1:**
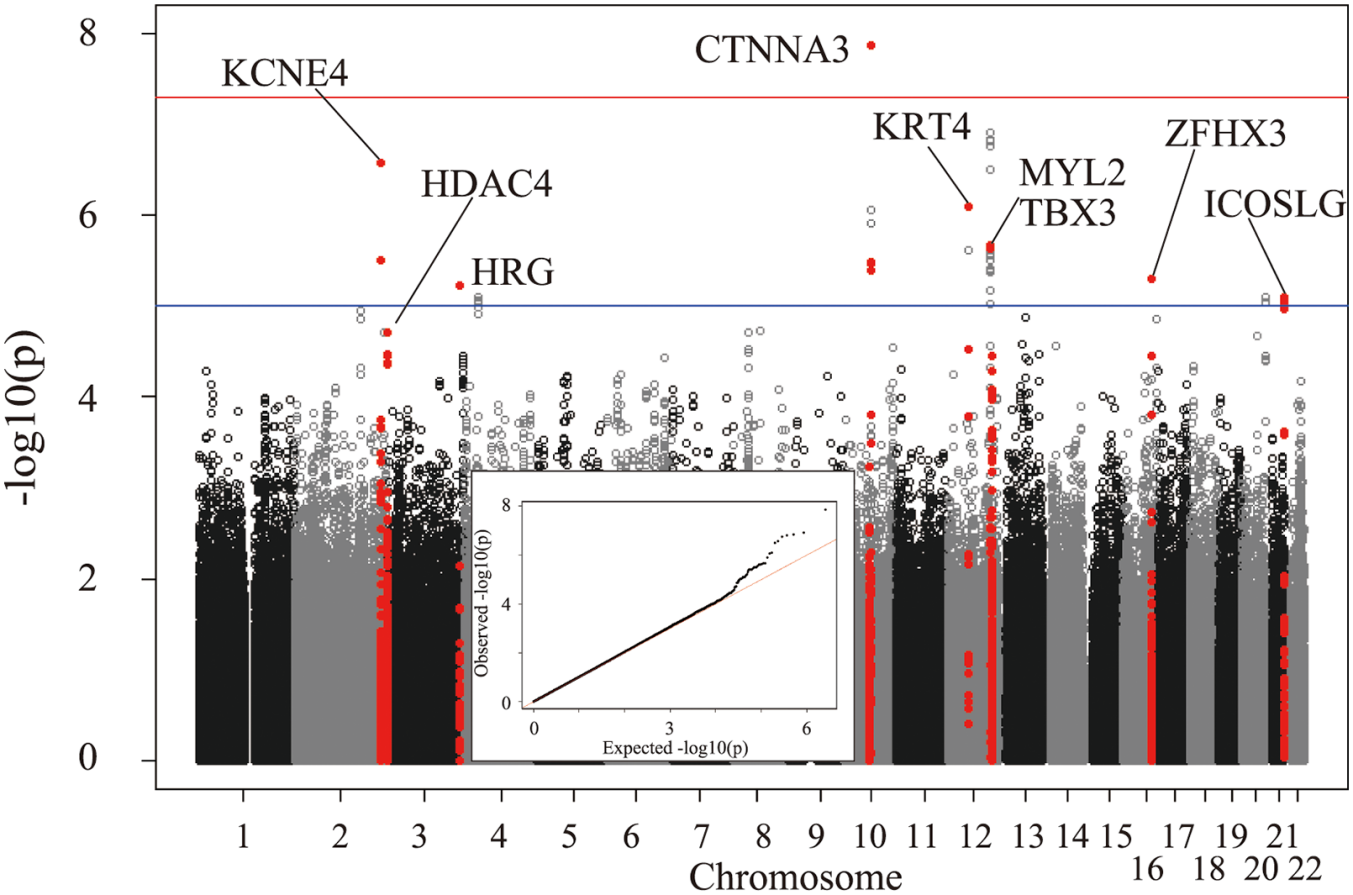
The results of GWAS on corrected electrocardiographic RV5 voltages. The Manhattan plots and quantile-quantile plots of genome-wide association results for RV5 from analysis of JPDSC datasets are shown.

After adjusting for non-genetic covariates, corrected electrocardiographic LV voltages (corrected RV5, corrected SV1, corrected RV5+SV1) were subjected to GWAS (Fig 1, S2 Fig). SNPs associated with RV5 and SV1 were not the same. Based on the Manhattan plots and quantile-quantile plots of genome-wide association results, we focused on loci associated with amplitude of the R wave in V5. The results of the GWAS on RV5 are summarized in Fig 1.

Using a pre-specified *P* value threshold of 1 X 10^−5^, we searched for genetic loci harbouring significant SNPs associated with RV5. SNPs below the threshold of *P* value < 10^−4^ and located in genes of interest were also included. We selected 13 SNPs based on a quality assessment survey of SNP genotyping and clarity of cluster (Table 2), and performed a replication study using an independent Japanese population obtained from the Nagahama Prospective Cohort for Comprehensive Human Bioscience (the Nagahama Study) (n = 2941). SNP rs7301743, which maps near *TBX3*, was replicated (Table 3).

**Table 2:** Results of GWAS for R wave in V5.

SNPID	CHR	POS	GENE	Minor/Major	MAF	Coefficient	P
rs2395983	2	224267984	KCNE4|SCG2	C/A	0.27	-0.066	2.63E-07
rs1921643	2	224294501	KCNE4|SCG2	A/G	0.251	-0.061	3.19E-06
rs1108519	2	240311883	HDAC4	G/A	0.182	0.064	3.39E-05
rs12613544	2	240327465	HDAC4|FLJ45964	G/A	0.172	0.067	1.94E-05
rs3733008	3	186387779	HRG	G/A	0.054	-0.113	6.08E-06
rs10430513	10	69410048	CTNNA3	A/G	0.259	-0.074	1.37E-08
rs10783539	12	53204757	KRT4	A/G	0.189	-0.073	8.14E-07
rs4766515	12	111308801	CCDC63	G/A	0.194	-0.077	1.24E-07
rs2301610	12	111353556	MYL2	G/A	0.207	-0.068	2.16E-06
rs7301743	12	115344561	TBX3|MED13L	G/A	0.408	-0.047	5.11E-05
rs12929452	16	72838680	ZFHX3	A/G	0.092	-0.091	5.12E-06
rs8131653	21	45644684	LOC100129890|ICOSLG	G/A	0.038	-0.131	7.93E-06
rs9977816	21	45653614	ICOSLG	G/A	0.039	-0.128	8.12E-06

**Table 3:** Replication study for R wave in V5.

SNP ID	CHR	POS	GENE	Minor/Major	MAF	Coefficient	P
rs2395983	2	224267984	KCNE4|SCG2	C/A	0.269	-0.001	9.35E-01
rs1921643	2	224294501	KCNE4|SCG2	A/G	0.245	-0.004	7.32E-01
rs1108519	2	240311883	HDAC4	G/A	0.193	-0.019	1.34E-01
rs12613544	2	240327465	HDAC4|FLJ45964	G/A	0.181	-0.017	2.12E-01
rs3733008	3	186387779	HRG	G/A	0.06	-0.033	1.13E-01
rs10430513	10	69410048	CTNNA3	A/G	0.262	0.003	7.98E-01
rs10783539	12	53204757	KRT4	A/G	0.189	0.018	1.69E-01
rs4766515	12	111308801	CCDC63	G/A	0.218	0.012	3.17E-01
rs2301610	12	111353556	MYL2	G/A	0.229	0.01	4.37E-01
rs7301743	12	115344561	TBX3|MED13L	G/A	0.427	-0.036	4.47E-04
rs12929452	16	72838680	ZFHX3	A/G	0.071	-0.009	6.59E-01
rs8131653	21	45644684	LOC100129890|ICOSLG	G/A	0.032	-0.036	5.84E-01
rs9977816	21	45653614	ICOSLG	G/A	0.032	-0.024	4.15E-01

### Meta-analysis of two Japanese datasets for possible novel associations

To obtain *P* values based on the results from two independent Japanese datasets, we performed a meta-analysis by the inverse variance method (Table 4). We observed a highly significant association of SNP rs7301743 in TBX3|MED13L with a 0.071 mV (95% CI, 0.038–0.11 mV) smaller voltage of the R wave in V5 per minor allele copy (P = 7.635 X 10^−8^). This SNP was located upstream of the coding region of TBX3 (S3 Fig).

**Table 4:** Meta-analysis for R wave in V5.

SNP	CHR	POS	GENE	Coefficient	SE	P
rs2395983	2	224267984	KCNE4|SCG2	-0.022	0.007	2.27E-03
rs1921643	2	224294501	KCNE4|SCG2	-0.03	0.009	4.75E-04
rs1108519	2	240311883	HDAC4	0.014	0.01	1.55E-01
rs12613544	2	240327465	HDAC4|FLJ45964	0.017	0.01	8.96E-02
rs3733008	3	186387779	HRG	-0.068	0.016	1.63E-05
rs10430513	10	69410048	CTNNA3	-0.033	0.009	1.43E-04
rs10783539	12	53204757	KRT4	-0.024	0.01	1.50E-02
rs4766515	12	111308801	CCDC63	-0.027	0.009	4.39E-03
rs2301610	12	111353556	MYL2	-0.024	0.009	8.12E-03
rs7301743	12	115344561	TBX3|MED13L	-0.041	0.008	7.64E-08
rs12929452	16	72838680	ZFHX3	-0.051	0.014	2.32E-04
rs8131653	21	45644684	LOC100129890|ICOSLG	-0.116	0.026	5.69E-06
rs9977816	21	45653614	ICOSLG	-0.079	0.02	7.00E-05

## Discussion

In healthy subjects from the JPDSC datasets, there was only a modest correlation between SV1 and RV5, both of which have been used as indices of LVH according to the Sokolow-Lyon criteria [11]. We have demonstrated that the voltage of the S wave in V1 (SV1), and that of the R wave in V5 (RV5) are differently affected by both non-genetic and genetic factors. Therefore, simply adding the S wave in V1 to the R wave in V5 may not be appropriate. We observed voltage attenuation of SV1 with advancing age, while the RV5 voltage was only modestly affected by age. The degree of obesity, as measured by BMI, was inversely correlated with the magnitude of SV1 in both sexes. By contrast, RV5 was less affected by BMI. Increased HR attenuated RV5, but not SV1, in both sexes. Comparison of the quantile–quantile plots (Q–Q plots) for SV1 and RV5 suggested that the RV5 was more strongly affected by genetic factors than the SV1. SNPs associated with RV5 and those associated with SV1 were not the same. The present GWAS study using the JPDSC dataset identified that only the IQCA1 gene locus was associated with both adjusted RV5 and SV1 voltages (p-values < 1 x 10^−3^).

We found that SNPs in the genes CTNNA3, MYL2, TBX3, ZFHX3, HDAC4, KCE4, and HERG were associated with the magnitude of the RV5 voltage. CTNNA3 encodes a protein belonging to the alpha-catenin family that plays a cell-cell adhesion role in cardiac muscle cells. Mutations in this gene are associated with arrhythmogenic right ventricular dysplasia [12]. MYL2 encodes a regulator protein of ventricular myosin ATPase activity and a mutation in this gene is associated with hypertrophic cardiomyopathy phenotype [13]. The zinc finger homeobox 3 (ZFHX3) gene encodes a transcription factor highly expressed in the heart and its genetic variants are associated with atrial fibrillation susceptibility [14]. HDAC4 encodes a class II histone deacetylase that modulates cardiac hypertrophic responses via epigenetic modifications [15]. KCNE4 and HERG encode potassium channels. Changes in potassium channel function are associated with cardiac hypertrophy [16]. Further studies are required to replicate these SNP associations.

Our GWAS and the subsequent validation study identified a novel association of SNP rs7301743[G], which maps near the T-box transcription factor *Tbx3* gene, with the R-wave voltage in the V5 lead of Japanese individuals. Members of the T-box (TBX) family of transcription factors have been identified as crucial players in various aspects of multi-chambered mammalian heart development, including cardiac lineage determination, chamber specification, valvuloseptal development, and diversification of the specialized conduction system in vertebrate embryos [17]. TBX1, TBX2, TBX3, TBX5, TBX18, and TBX20 are expressed in the mammalian heart, and all exhibit complex temporal and spatial regulation in developing cardiac structures. TBX1 and TBX20 promote development of the outflow tract and right ventricular myocardium, while TBX5 promotes development of left ventricular myocardium. TBX2 and TBX3 trigger development of endocardial cushions, the precursors of the atrioventricular septum, valves, and conduction system, while suppressing development of the working ventricular myocardium.

Meta-analysis of two independent Japanese datasets demonstrated a highly significant association of SNP rs7301743 in TBX3|MED13L with a 0.071 mV (95% CI, 0.038–0.11 mV) shorter amplitude of the R wave in the V5 lead per minor allele copy (P = 7.635 X 10^−8^). Since TBX3 is able to suppress a large panel of working myocardial genes in the fully differentiated working myocardium [18], we speculate that genetic mutations alter temporal and spatial regulation of TBX3 expression, thereby affecting LV free wall mass under steady-state conditions or changing LVH susceptibility.

Our approach has resulted in the detection of novel genetic factors associated with LV mass, and may be compared with the widely used method of detecting LVH by fixed voltage criteria. Differences between this study and previous studies are in part due to selection bias. Previous studies enrolled more pathologically extreme LVH encountered in a hospital-based cohort. This study is based on a primarily healthy population.

The present study has some limitations. First, our retrospective study evaluated left ventricular mass by ECG because it was a standard diagnostic method. Estimates by ECG, however, are less accurate than estimates by cardiac MRI. Second, meta-analysis of two independent Japanese datasets demonstrated that association of SNPs in TBX3 with the RV5 voltage variation was the most significant, however, did not reach the genome-wide significance threshold (P ≤ 5 × 10^−8^). Third, in GWAS studies, associated SNPs are often in the non-coding region. Whether the associated SNPs really affect the phenotype (in this case, LVH) is often argued but confirmatory explanation is often difficult. If there are eQTL data for human heart, we may able to test whether the associated SNP changes the mRNA of a gene but this was not possible in our study.

## Supporting Information

S1 Fig We used the multiple linear regression analysis to estimate clinical factors accounting for individual variation in LVH parameters, after removing outliers defined as the values that are either higher or lower than 4*SD from the mean. (DOCX)

S2 Fig Regional plot for R wave in V5 focused to TBX3. (DOCX)

S3 Fig The Manhattan plots and quantile-quantile plots of genome-wide association results for SV1 and RV5+SV1. (DOCX)

S1 Table Multiple linear regression analysis to estimate clinical factors accounting for individual variation in LVH parameters. (DOCX)
