# Supplementary material for: Genome-Wide Association Study of Absolute QRS Voltage Identifies Common Variants of TBX3 as Genetic Determinants of Left Ventricular Mass in a Healthy Japanese Population
Source: PLoS One. 2016 May 19;11(5):e0155550. doi: 10.1371/journal.pone.0155550 (PMC4873129; doi:10.1371/journal.pone.0155550)
Supplement: S1 Fig — (DOCX) [file pone.0155550.s001.docx]

**S1 Fig. We used the multiple linear regression analysis to estimate clinical factors accounting for individual variation in LVH parameters, after removing outliers defined as the values that are either higher or lower than 4*SD from the mean.**

(A) Original

| RV5 | SV1 | RV5+SV1 |
| --- | --- | --- |
| 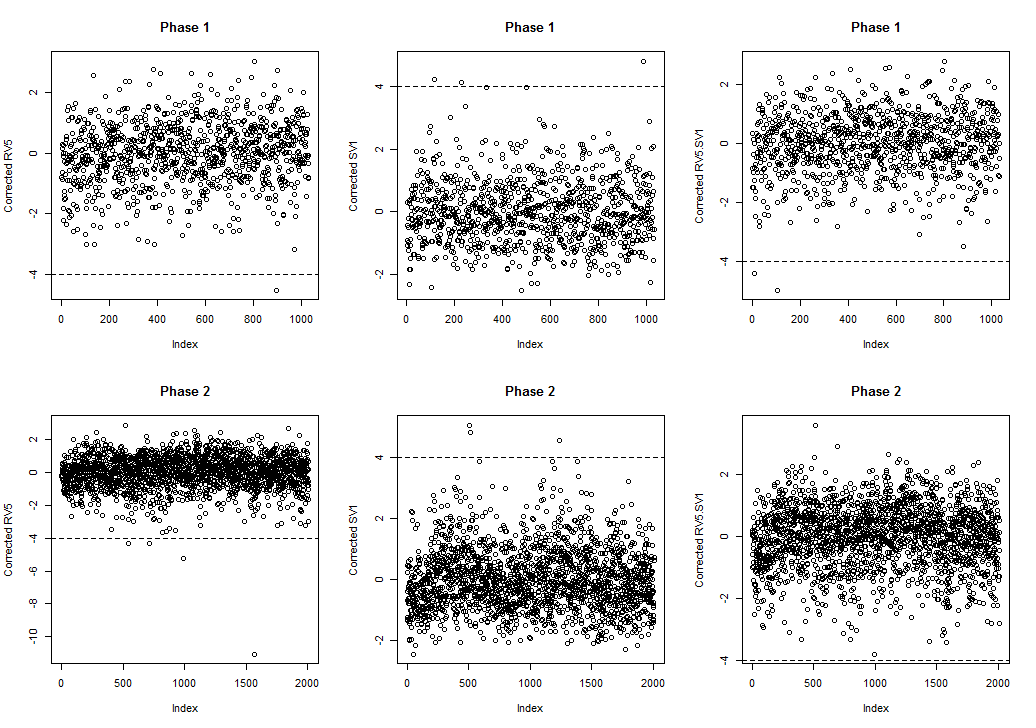 | | |

(B) Removed outliers

| RV5 | SV1 | RV5+SV1 |
| --- | --- | --- |
| 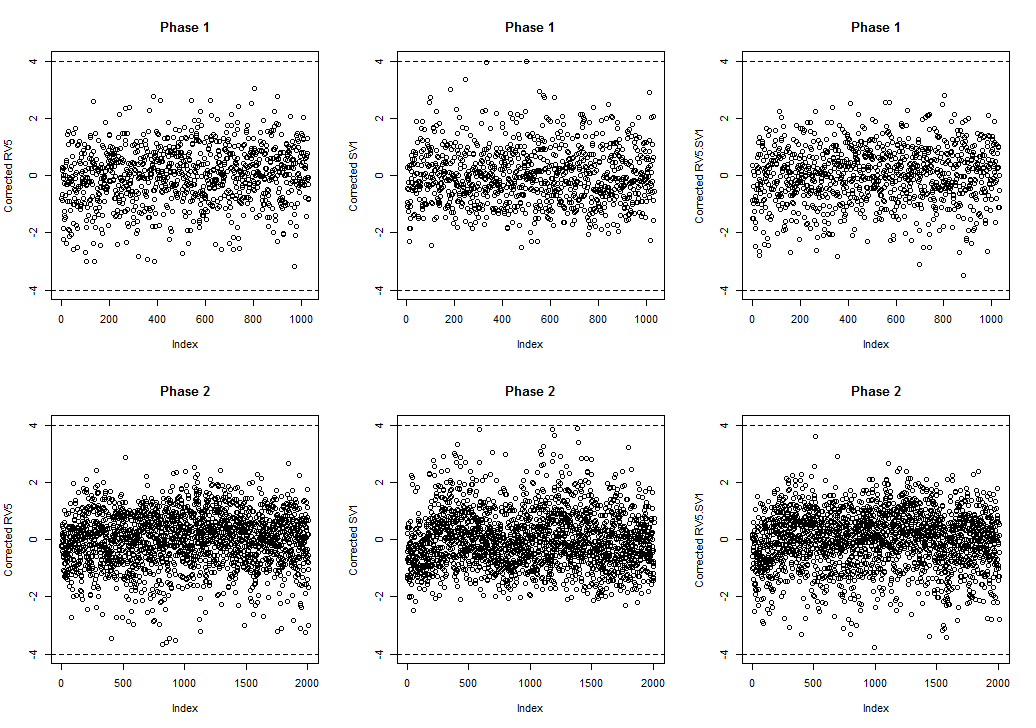 | | |
