# Supplementary material for: Genome-Wide Association Study of Absolute QRS Voltage Identifies Common Variants of TBX3 as Genetic Determinants of Left Ventricular Mass in a Healthy Japanese Population
Source: PLoS One. 2016 May 19;11(5):e0155550. doi: 10.1371/journal.pone.0155550 (PMC4873129; doi:10.1371/journal.pone.0155550)

**S2 Fig. The Manhattan plots and quantile-quantile plots of genome-wide association results for RV5 (upper left), SV1 (Upper right) and RV5+SV1 (lower left).**


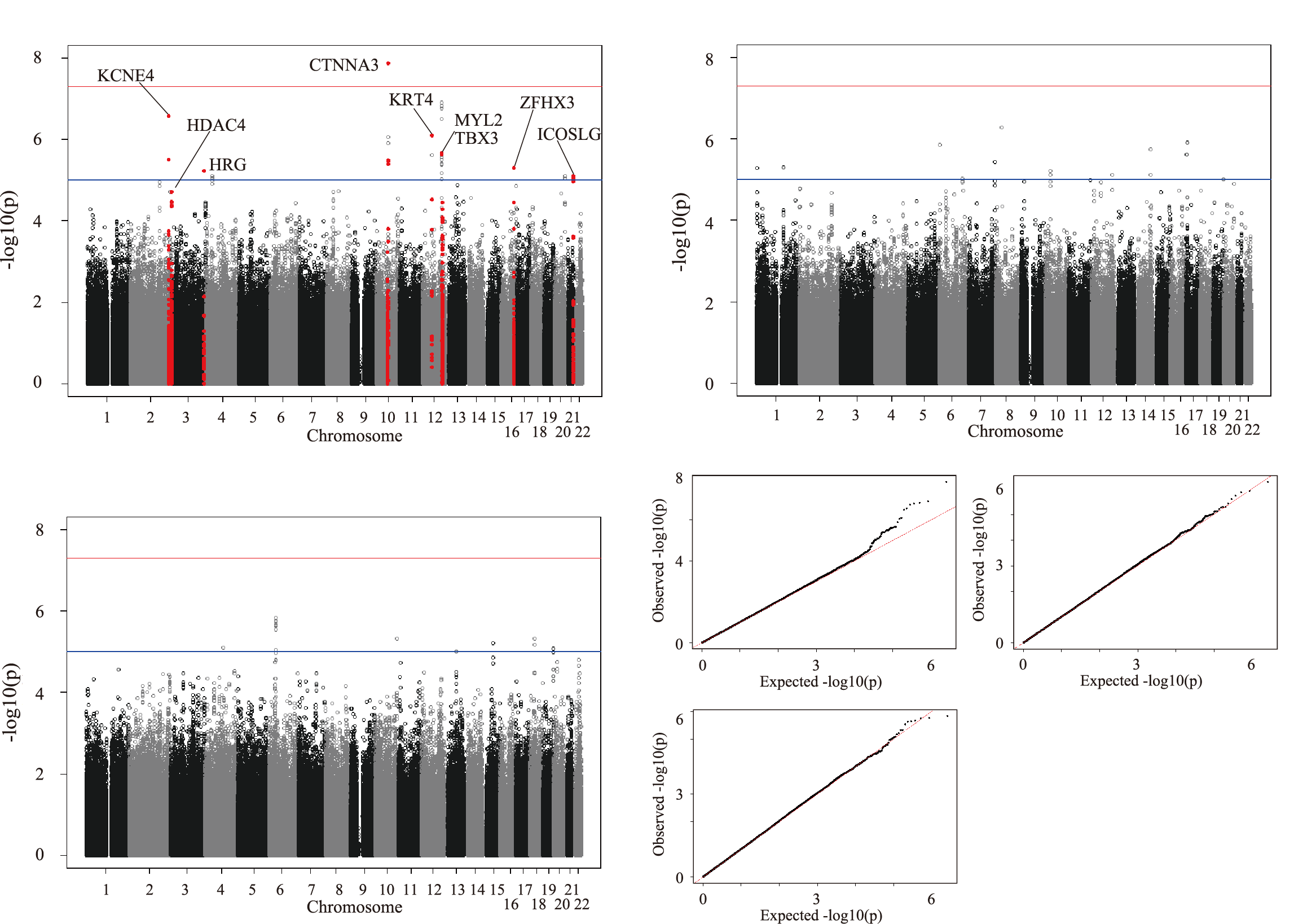

Supplement: S2 Fig — (DOCX) [file pone.0155550.s002.docx]
