# Supplementary figures and images for: Genome-Wide Association Study of Absolute QRS Voltage Identifies Common Variants of TBX3 as Genetic Determinants of Left Ventricular Mass in a Healthy Japanese Population
Source: PLoS One. 2016 May 19;11(5):e0155550. doi: 10.1371/journal.pone.0155550 (PMC4873129; doi:10.1371/journal.pone.0155550)

**S3 Fig. Regional plot for R wave in V5 focused to TBX3.**

| 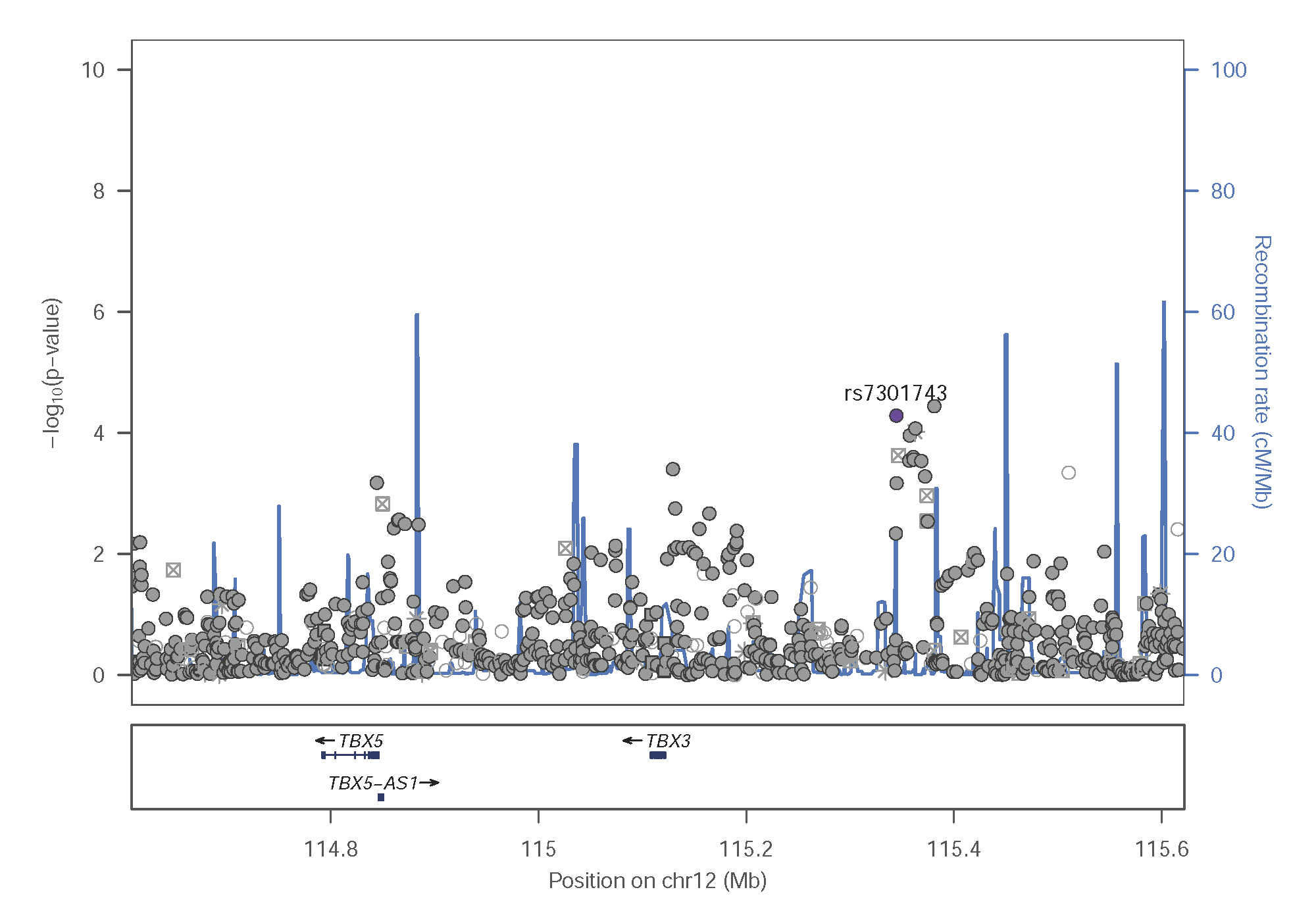 |
| --- |

Supplement: S3 Fig — (DOCX) [file pone.0155550.s003.docx]
