# Supplementary material for: Genome-Wide Association Study of Absolute QRS Voltage Identifies Common Variants of TBX3 as Genetic Determinants of Left Ventricular Mass in a Healthy Japanese Population
Source: PLoS One. 2016 May 19;11(5):e0155550. doi: 10.1371/journal.pone.0155550 (PMC4873129; doi:10.1371/journal.pone.0155550)
Supplement: S1 Table — (DOCX) [file pone.0155550.s004.docx]

**S1 Table. Multiple linear regression analysis to estimate clinical factors accounting for individual variation in LVH parameters**

(A) R wave in V5

| Variable | Phase1 |  | Phase2 |  | Meta analysis | |
| --- | --- | --- | --- | --- | --- | --- |
|  | Coefficient | Wald P | Coefficient | Wald P | Coefficient | P |
| (Intercept) | 0.480 | 2.939E-01 | 0.605 | 8.309E-02 | 0.559 | 4.386E-02 |
| Age | 0.002 | 1.815E-02 | 0.000 | 8.104E-01 | 0.001 | 1.677E-01 |
| Gender:Male | 0.253 | 6.200E-26 | 0.259 | 1.157E-45 | 0.257 | 0.000E+00 |
| log(BMI) | -0.093 | 3.100E-01 | 0.061 | 3.151E-01 | 0.014 | 7.834E-01 |
| systole | 0.002 | 1.064E-01 | 0.004 | 3.677E-07 | 0.004 | 2.147E-07 |
| diastole | 0.004 | 6.680E-02 | -0.001 | 5.292E-01 | 0.000 | 7.562E-01 |
| log(HR) | -0.238 | 6.951E-04 | -0.300 | 2.602E-08 | -0.277 | 7.790E-11 |
| K | 0.041 | 2.226E-01 | -0.028 | 9.082E-02 | -0.015 | 3.329E-01 |
| Ca | 0.035 | 2.289E-01 | 0.034 | 1.544E-01 | 0.034 | 6.220E-02 |
| PCA1 | 0.270 | 8.378E-01 | 0.374 | 3.757E-01 | 0.364 | 3.648E-01 |
| PCA2 | -0.173 | 7.792E-01 | -0.716 | 1.887E-01 | -0.477 | 2.416E-01 |

(B) S wave in V1

| Variable | Phase1 |  | Phase2 |  | Meta analysis | |
| --- | --- | --- | --- | --- | --- | --- |
|  | Coefficient | Wald P | Coefficient | Wald P | Coefficient | P |
| (Intercept) | 2.768 | 2.670E-05 | 2.158 | 8.913E-07 | 2.346 | 1.170E-10 |
| Age | -0.007 | 8.487E-08 | -0.007 | 2.968E-10 | -0.007 | 1.110E-16 |
| Gender:Male | 0.135 | 6.231E-05 | 0.134 | 2.447E-09 | 0.134 | 5.326E-13 |
| log(BMI) | -0.749 | 1.833E-08 | -0.461 | 2.256E-09 | -0.533 | 8.882E-16 |
| systole | 0.001 | 7.707E-01 | 0.006 | 6.998E-08 | 0.005 | 9.864E-07 |
| diastole | 0.007 | 1.535E-02 | -0.003 | 6.499E-02 | -0.001 | 5.190E-01 |
| log(HR) | -0.034 | 7.340E-01 | -0.070 | 3.008E-01 | -0.059 | 2.943E-01 |
| K | -0.036 | 4.553E-01 | -0.013 | 5.370E-01 | -0.017 | 3.873E-01 |
| Ca | 0.047 | 2.590E-01 | 0.029 | 3.261E-01 | 0.035 | 1.457E-01 |
| PCA1 | -1.476 | 4.354E-01 | 0.443 | 4.024E-01 | 0.304 | 5.510E-01 |
| PCA2 | 0.730 | 4.090E-01 | 0.576 | 3.985E-01 | 0.634 | 2.407E-01 |

(C) The sum of RV5 and SV1

| Variable | Phase1 |  | Phase2 |  | Meta analysis | |
| --- | --- | --- | --- | --- | --- | --- |
|  | Coefficient | Wald P | Coefficient | Wald P | Coefficient | P |
| (Intercept) | 1.711 | 1.417E-05 | 1.675 | 1.649E-08 | 1.688 | 8.480E-13 |
| Age | -0.001 | 1.457E-01 | -0.003 | 1.486E-04 | -0.002 | 1.293E-04 |
| Gender:Male | 0.201 | 1.681E-22 | 0.202 | 3.724E-39 | 0.201 | 0.000E+00 |
| log(BMI) | -0.353 | 8.883E-06 | -0.157 | 2.422E-03 | -0.216 | 6.097E-07 |
| systole | 0.001 | 1.976E-01 | 0.005 | 2.066E-11 | 0.004 | 1.369E-10 |
| diastole | 0.005 | 5.927E-03 | -0.001 | 3.138E-01 | 0.000 | 6.923E-01 |
| log(HR) | -0.156 | 9.546E-03 | -0.220 | 1.413E-06 | -0.197 | 5.811E-08 |
| K | 0.016 | 5.842E-01 | -0.023 | 1.092E-01 | -0.015 | 2.319E-01 |
| Ca | 0.033 | 1.903E-01 | 0.018 | 3.686E-01 | 0.024 | 1.281E-01 |
| PCA1 | -0.357 | 7.525E-01 | 0.441 | 2.173E-01 | 0.369 | 2.795E-01 |
| PCA2 | 0.078 | 8.826E-01 | -0.209 | 6.508E-01 | -0.085 | 8.071E-01 |
